# Bacterial Vaccinations in Patients with Chronic Obstructive Pulmonary Disease

**DOI:** 10.3390/vaccines12020213

**Published:** 2024-02-18

**Authors:** Dóra Paróczai, Katalin Burian, Andras Bikov

**Affiliations:** 1 Department of Medical Microbiology, University of Szeged, H-6720 Szeged, Hungary; paroczai.dora@med.u-szeged.hu (D.P.); burian.katalin@med.u-szeged.hu (K.B.); 2 Albert Szent-Györgyi Health Center, Department of Pulmonology, University of Szeged, H-6720 Szeged, Hungary; 3 Manchester University NHS Foundation Trust, Wythenshawe Hospital, Manchester M23 9LT, UK; 4 Division of Immunology, Immunity to Infection and Respiratory Medicine, University of Manchester, Manchester M13 9PL, UK

**Keywords:** COPD, vaccination, bacteria

## Abstract

Chronic obstructive pulmonary disease (COPD) is a frequent, often progressive, chronic disease of the lungs. Patients with COPD often have impaired immunity; therefore, they are prone to chest infections, such as pneumonia or bronchitis. Acute exacerbations of COPD are major events that accelerate disease progression, contributing to its symptoms’ burden, morbidity, and mortality. Both pneumonia and acute exacerbations in COPD are caused by bacteria against which there are effective vaccinations. Although the number of randomised controlled studies on bacterial vaccinations in COPD is limited, national and international guidelines endorse specific vaccinations in patients with COPD. This review will summarise the different types of vaccinations that prevent pneumonia and COPD exacerbations. We also discuss the results of early phase studies. We will mainly focus on *Streptococcus pneumoniae*, as this bacterium was predominantly investigated in COPD. However, we also review studies investigating vaccinations against *Haemophilus influenzae*, *Moraxella catarrhalis*, and *Bordetella pertussis*.

## 1. Introduction

Chronic obstructive pulmonary disease (COPD) affects approximately 10% of the global adult population, stemming from a combination of environmental triggers (such as cigarette smoke and indoor pollution) and genetic predisposition. It is characterized by progressive, fixed airflow limitation, leading to chronic breathlessness and productive cough [1]. This typically progressive condition is occasionally punctuated by sudden exacerbations, significant events associated with reduced life expectancy [2], often triggered by bacterial infections [3].

Patients with COPD exhibit aberrant airway inflammation, marked by an influx of neutrophils and macrophages [4], alongside impaired antimicrobial responses [5]. Alveolar macrophages from COPD patients demonstrate deteriorating phagocytic functions against common bacteria, such as *Streptococcus pneumoniae* (*S. pneumoniae*), *Haemophilus influenzae* (*H. influenzae*), and *Moraxella catarrhalis* (*M. catarrhalis*) [6]. Interestingly, these bacteria induce a milder inflammatory response in COPD patients [7].

Medications, particularly inhaled corticosteroids, commonly prescribed for COPD patients with eosinophilic airway inflammation and frequent exacerbations, may reduce exacerbation rates but potentially increase the risk of chest infections [8,9], depending on factors such as molecule type, formulation size, and dosage [10]. Additionally, prophylactic antibiotics such as azithromycin can prevent exacerbations [11].

Given the susceptibility of COPD patients to bacterial chest infections, including pneumonia and bronchitis, vaccinations against common pathogens are recommended [1,12]. This review aims to summarize evidence on the effectiveness of vaccinations in preventing chest infections and exacerbations in COPD.

## 2. The Importance of Bacterial Vaccines in COPD Care

Bacterial culturing techniques and next-generation sequencing have confirmed the significant impact of the lung microbiome on disease severity and exacerbations in COPD. Unlike in healthy individuals, COPD lungs exhibit higher pathogenic bacterial colonization and reduced diversity of normal flora [13]. Inhaled noxious particles, such as tobacco smoke, elevate the risk of bacterial infections by disrupting mucociliary clearance, impairing airway epithelium, and altering microbiome composition [14]. Additionally, during infectious exacerbations, bacteria such as *H. influenzae*, combined with antibiotic use, can further disrupt the lung microbiome and escalate the risk of exacerbations [15]. Patients with COPD are particularly susceptible to respiratory pathogens due to an increased expression of cell-adhesion molecules such as the platelet adhesion factor receptor or intercellular adhesion molecule 1 (ICAM1) in their airways, heightening the risk for *S. pneumoniae* and *H. influenzae* infections [16,17]. Hence, it is evident why approximately two-thirds of COPD exacerbations are associated with infectious pathogens or bacterial colonization [18,19]. The surge in COVID-19 vaccination efforts has underscored the importance of evaluating the clinical efficacy of current vaccines in respiratory diseases. As part of exacerbation prevention strategies, vaccination against bacteria such as *S. pneumoniae* is incorporated into both national and international recommendations [1].

Despite understanding the primary effects of current vaccinations, clinical efficacy and vaccination rates against exacerbations can raise some concerns. In this session, we summarise traditional and emerging vaccinations targeting bacteria implicated in potentially exacerbating COPD.

## 3. Pneumococcal Vaccination

### 3.1. Serotypes and Vaccine Options

To date, 98 different serotypes of *S. pneumoniae* were identified, with 23 of them being the major cause of invasive pneumococcal diseases [20,21]. The most common isolate is serotype 14, followed by 1, 5, 6A, 6B, 19F, 23F serotypes, which can be found in more than 50% of patients [22]. However, the widespread use of vaccination caused a serotype replacement and capsular switching, increasing the chance that a relevant serotype is not included in the vaccine formulations [23]. The non-vaccine serotypes are highly invasive and have showed high antibiotic resistance, thus increasing the need for new vaccine development [24].

Pneumococcal vaccines are primarily capsular polysaccharide antigens in either unconjugated (PPSV23) or conjugated form (PCVs), along with protein-based vaccines, live attenuated or killed whole-cell vaccines, and promising nanovaccines [25].

### 3.2. PPSV23 and PCVs

The first pneumococcal vaccine, a 23-valent vaccine (PPSV23) contains capsular polysaccharide antigens of serotypes 1, 2, 3, 4, 5, 6B, 7F, 8, 9N, 9V, 10A, 11A, 12F, 14, 15B, 17F, 18C, 19A, 19F, 20, 22, 23F, 33F and has been introduced to clinical practice since 1983. The vaccine is recommended for individuals above age 65 or between age 2 and 64, with comorbidities [26]. Although PPSV23 covers a wide range of serotypes that are responsible for the majority of invasive pneumococcal diseases, and clinical trials revealed that it can reduce the severity of pneumonia, it is less effective for the prevention of pneumonia [27]. Another meta-analysis showed that despite some evidence of short-term protection, the efficacy of PPSV23 against community-acquired pneumonia (CAP) in the general population, immunocompromised individuals, and individuals with potential risk factors is inconsistent [28]. Additionally, PPSV23 showed poor immunogenicity in patients with immunosuppressive conditions, such as transplantation or HIV [29,30]. In addition, the vaccine is not suitable to trigger long-term immunological memory in children, as PPSV23 requires T-cell independent immunity. Therefore, children show a lower response to this vaccine [31,32]. In the case of adults, PCVs induced a more favourable, superior functional antibody response, compared to PPSV23 [33]. Dransfield et al. confirmed that in patients with COPD, the conjugated vaccine (PCV7) was superior to PPSV23 alone, and that induced immunogenicity can persist for 2 years post-vaccination [34,35]. The PCV vaccine era was started with the introduction of PCV7 in 2000, following PCV10 and PCV13 [36,37]. Despite the use of PPSV23, CDC reported that the majority of the isolated serotypes of *S. pneumoniae* from immunocompromised patients are not included in the PPSV23 [38]. This recognition elicited the need to broaden the coverage of PCVs. PCV13 was developed to cover *S. pneumoniae* serotype 1, 3, 4, 5, 6A, 6B, 7F, 9V, 14, 18C, 19A, 19F, and 23F, and it was approved for use in adults [38]. PCV13 was also safely used in the elderly (>65 age), showing good immunogenicity [39].

### 3.3. Clinical Efficacy of PPSV23 in Chronic Respiratory Conditions

In 2010, the first systematic review that included seven randomized controlled trials (RCTs) revealed controversial results, as using PPSV23 did not reduce the prevalence of pneumonia in patients with COPD compared to control individuals (odds ratio (OR) and 95% confidence interval (CI) of 0.72 and 0.51 to 1.01). The reduction in the likelihood of acute COPD exacerbations, investigated by only two studies on 216 people, was not statistically significant (OR 0.58; 95% CI 0.30 to 1.13). In addition, there were no statistically significant effects on reduction in hospital admissions [40].

In the study by Steentoft et al., 49 patients with COPD were randomly assigned to four groups, and the authors investigated whether steroid use and/or vaccination can influence the clinical variables, including hospital admission or exacerbation rate. They found that there were no differences in the prevalence of pneumonia, exacerbations, and hospitalization between the vaccinated and control patients [41]. An update of the Cochrane analysis in 2017 included 12 RCTs, and only one study compared the effectiveness of PCV7 with that of PPSV23. The meta-analysis showed that the general risk of CAP was significantly reduced in vaccinated patients (OR 0.62, 95%CI 0.43 to 0.89), but it was not observed in the case of pneumococcal pneumonia (OR 0.26, 95%CI 0.05 to 1.31). Although vaccinations (mainly with PPSV23) significantly reduced the likelihood of COPD exacerbations (OR 0.60, 95% CI 0.39 to 0.93), the risk of hospital admission and mortality were not different between the control and vaccinated participants [42]. A prospective randomised trial by Alfageme et al. showed that the PPSV23 vaccine efficacy was only 24% across all patients. However, the vaccine was effective in preventing CAP only in patients <65 ages and having severe airflow obstruction (FEV1 < 40%). There were no differences in all-cause mortality, but the study had gender imbalance, so the applicability of the trial raised some questions [43]. Indeed, the effectiveness of PPSV23 has been questioned for the elderly or at-risk populations for years. A prospective, multicentre, double-blind, randomised, placebo-controlled trial including 339 patients in the vaccine group and 352 in the placebo group revealed that the 23-valent pneumococcal polysaccharide vaccine did not prevent pneumonia in patients aged between 50 and 85 [44]. Another trial also did not observe a positive effect on altering the risk of pneumococcal pneumonia in patients >65 years, but the vaccine was associated with a significant reduction in the risk of pneumococcal bacteraemia [45].

Regarding the efficacy of PPSV23, long-standing immune responses were detected in healthy adults: IgG and functional antibody levels remained above the baseline for 5–10 years. However, similar studies with COPD participants are lacking [46]. So far, one investigation has given evidence on the correlation between the reduced quantity or functionality of pneumococcal IgG antibodies and frequent COPD exacerbations, suggesting that higher baseline IgG levels were predictive of fewer and less severe exacerbations. Moreover, from the tested 23 serotypes, specific IgG for five serotypes (5, 19F, 10A, 15B, and 19A) proved to be significant predictors for COPD exacerbations. They also demonstrated that patients having more exacerbations had an altered function of pneumococcal antibodies and an impaired opsonisation ability [47].

Table 1 summarizes the key clinical studies that assess the outcomes of pneumococcal vaccinations in individuals with chronic obstructive pulmonary disease (COPD).

### 3.4. Co-Administration of PPSV23 and Antiviral Vaccines

Clinicians sought to enhance the efficacy of the pneumococcal vaccine and investigated the effect of the co-administration of PPSV23 and influenza vaccines on COPD exacerbations. A randomized, controlled study suggested that COPD patients receiving both the influenza and PPSV23 vaccines are at a significantly lower risk for exacerbations, compared to those who received only one vaccine. However, this effect lasted for 1 year, and during the second year there were no significant beneficial effects on exacerbations [48]. An earlier retrospective study also suggested that administering both pneumococcus and influenza vaccines to patients with COPD resulted in a 63% (95% CI 29–80) reduction in pneumonia hospitalizations and an 81% (95% CI 68–88) reduction in deaths, compared to unvaccinated patients [49]. Another study included patients older than 65 years, and significant reductions in hospital admissions and hospitalizations for invasive pneumococcal disease and pneumococcal pneumonia were observed in people who also received the pneumococcal vaccine. Efficacy against heart failure has also been observed in subjects who received the pneumococcal vaccine. During flu seasons, a reduction in all-cause mortality was also observed in all age groups [50]. In line with this observation, a recent study supported the favourable effects of influenza and PPSV23 vaccinations, resulting in a reduced frequency of exacerbation in the following year, with an OR of 1.06 (95% CI: 0.84–1.34) for PPSV23 alone, and an OR of 2.37 (95% CI: 1.39–4.08) for patients who were administered both vaccines [51]. In 2022, similar results were obtained by Li et al. Out of 474 enrolled patients, 109 received trivalent influenza vaccines, 69 patients were administered PPSV23, and 296 participants received both. The vaccine efficacy for exacerbations was 54% for PPSV23 alone and 72% for the combination. The additive impact was estimated as a corresponding effectiveness of 73% in reducing pneumonia and 69% in reducing hospitalization rate [52].

In an open-label, randomized, controlled trial, 1152 adults aged over 18 years with no prior history of pneumococcus, influenza, or SARS-CoV-2 vaccination were enrolled to assess the safety and immunogenicity of co-administering an inactivated SARS-CoV-2 vaccine (Sinopharm BBIBP-CorV), a quadrivalent split-virion inactivated influenza vaccine (IIV4), and a 23-valent pneumococcal polysaccharide vaccine (PPSV23). The seroconversion rate of SARS-CoV-2 and influenza neutralising antibodies in the SARS-CoV-2 + IIV4/PPSV23 group were comparable with those of the groups who did not receive the combination. Similarly, the immunogenicity of the SARS-CoV-2 + IIV4/PPSV23 group, as indicated by *S. pneumoniae*-specific IgG levels, was not inferior to that of the IIV4/PPSV23 group. The data revealed that the co-administration of inactivated SARS-CoV-2 vaccine with PPSV23 and influenza vaccine is well-tolerated and trigger comparable immune responses [53].

**Table 1:** The primary clinical investigations aimed at assessing the outcomes of pneumococcal vaccinations in individuals diagnosed with COPD.

Reference	Vaccine Type	Study Design	Patients (n)	Main Clinical Outcomes
Davis et al. [54] (1987)	PPSV14	Prospective, randomized PPSV14 vs. control	103	No differences in all-cause mortality and pneumonia risk at 12 and 24 months were observed.
Alfageme et al. [43] (2006)	PPSV23	RCT, PPSV23 vs. control	596	PPSV23 is efficient in averting CAP only in COPD patients under the age of 65 and with severe airflow obstruction.
Steentoft et al. [41] (2006)	PPSV23	Prospective, randomized PPSV23 vs. control	49	60–78% of vaccinated patients exhibited an increase in antibody levels. No disparities were noted in clinical parameters, such as pneumonia incidence, exacerbations, hospital admissions, escalation in steroid or β-agonist use, or antibiotic consumption.
Furumoto et al. [48] (2008)	PPSV23, trivalent influenza vaccine (IV)	Prospective, randomized PPSV23+IV vs. IV	191	The likelihood of infectious exacerbations was reduced in both groups, and the combined effect of PV and IV was observed during the initial year.
Dransfield et al. [34] (2009)	PPSV23, PCV7	Prospective, randomized PCV7 vs. PPSV23	120	PCV7 induced superior immune responses compared to PPSV23 1-month post-vaccination. Older age and a history of PPSV23 infection reduced vaccine responsiveness.
Dransfield et al. [35] (2012)	PPSV23, PCV7	Prospective, randomized PCV7 vs. PPSV23	181	PCV7 induced stronger functional antibody responses compared to PPSV23, lasting 2 years.
Kostinov et al. [55] (2014)	PPSV23	Prospective, randomized PPSV23 vs. control	200	PPSV23 group experienced fewer exacerbations, hospital admissions, and days of disability within the first year of the study compared to control participants.
Ignatova et al. [56] (2021)	PPSV23, PCV13	Prospective, observational	302	By the fifth year after vaccination, 47% of patients in the PPV23 group experienced pneumonia, compared to only 3.3% in the PCV13 group. COPD exacerbations were reported in 81.3% versus 23.6% of patients. Both vaccines exhibited comparable clinical effects in the first-year post-vaccination, but only PCV13 demonstrated sustained clinical efficacy.

### 3.5. Clinical Efficacy of PCVs in COPD

Pneumococcal conjugate vaccines were intended for children firstly in a form of a 7-valent diphtheria toxin-conjugated pneumococcal polysaccharide vaccine to induce more potent immune responses and decrease the frequency of invasive pneumococcal diseases [57]. Although PCV7 is not used for adults in clinical practice, early research demonstrated that PCV7 was associated with enhanced functional anti-pneumococcal antibodies and better immune response in a dose-dependent manner [58]. If we speculate about the quality of immune responses when comparing polysaccharide and conjugated vaccines, there is strong evidence that the PPSV23 vaccine is limited to B-cell stimulation and triggers less immune response, resulting in the deficit of the immune memory [59,60]. Data about the B-cell-specific responses in adult are still controversial, with some studies suggesting that there are no significant differences between PCVs and PPSV23 in elderly patients [61,62]. The Centers for Disease Control (CDC) reported that only less than 25% of the total number of invasive pneumococcal disease cases in the immunocompromised patients were caused by PPSV23-covered *S. pneumoniae* serotypes (8, 9N, 10A, 11A, 12F, 15B, 17F, 18C, 19A, 20, 22F, and 33F). PCV13, which was licensed in 2011, provides coverage for *S. pneumoniae* serotypes 1, 3, 4, 5, 6A, 6B, 7F, 9V, 14, 18C, 19A, 19F, and 23F and was recommended for adults aged >50 years. Clinical recommendations were based on immunogenicity and safety studies that showed enhanced or comparable opsonophagocytic activity and antibody titres compared to the responses elicited by PPSV23 in this population [38]. Another study provided similar evidence, namely that PCV13 is safe and provoked sufficient immune responses in the elderly aged >65 years [39]. Recently, CDC recommended PCV13 vaccination in adults aged ≥65 years based on clinical decision-making because some patients can be at increased risk for exposure to PCV13 serotypes and have higher risk for developing pneumococcal disease as a result of underlying medical conditions [26]. Although there are no further studies dedicated to the immunocompromised patients regarding the long-term immunogenicity of PCV13, the latter is supported by a study with HIV-positive adults, which suggests a durable, long-term immune response to PCV7 against invasive pneumococcal diseases, induced by one or two doses of PCV7 [63].

A large, randomized, double-blind, placebo-controlled trial (Community-Acquired Pneumonia Immunization Trial in Adults (CAPiTA)), which involved 84,496 adults aged 65 years or older, evaluated the efficacy of PCV13 in participants with chronic medical conditions, being at higher risk of pneumococcal disease. The study demonstrated that 49 patients in the PCV13 and 90 of the control group had CAP (vaccine efficacy of 45.6%; 95.2% CI, 21.8 to 62.5). Non-bacterial and non-invasive CAP was observed in 33 persons in the PCV13 group, and in 60 persons in the control group (vaccine efficacy of 45.0%; 95.2% CI, 14.2 to 65.3). The vaccine efficacy was continuous during the study, with a mean follow-up time of 3.97 years [64]. A post-hoc analysis of the CAPiTA study dedicated to chronic medical conditions, including COPD, showed that 82.7% of vaccine serotype CAP occurred in participants with underlying comorbidities, with around 10% of this group having chronic lung diseases. The vaccine efficacy was 40.3% (95.2% CI: 11.4%, 60.2%) in the at-risk population, the average duration of immune protection being 3.95 years [65]. The CAPiTA study suggested that PCV13 has long-term efficacy in elderly aged >65 years, with or without chronic medical conditions, such as COPD. Thus, PCV13 is included in the vaccine recommendation protocols across Europe.

Although there is evidence on the efficacy of PCV13 and PPSV23, the comparative analysis of their immunogenicity in COPD is still lacking. The first investigations compared PCV7- and PPSV23-vaccinated COPD subjects, evaluated post-vaccination antibody levels and the opsonophagocytic killing index (OPK), according to vaccine type and *S. pneumoniae* serotypes. It was found that post-vaccination, the IgG levels at 1 month were significantly higher in the PCV7 group for all seven vaccine serotypes, compared to PPSV23. Patients with COPD were able to mount sufficient functional immune responses to PPSV23 and PCV7. However, OPK was significantly higher in the case of PCV7-vaccinated patients, suggesting a greater immune defence against pneumococcal diseases [34]. A study by Dransfield et al. investigated the long-term immunogenicity (at 1 and 2 years) of PCV7 and PV23 in 181 COPD patients. The study reported a statistically greater induced OPK for 5 serotypes after PCV7 vaccination at 1 year and for 4 serotypes at 2 years. OPK was not affected by any clinical factors, such as FEV1 or prior vaccination status. Both vaccines induced high IgG antibody levels that persisted for 2 years. However, for PCV7, these levels were greater than for PPSV23. This study provided information about a comparative effectiveness of PCV and PPSV23, raising the issue of the superiority of PCVs in COPD patients [35].

Only one prospective observational cohort study was conducted to assess the long-term vaccine efficacy of PPSV23 and PCV13 in patients with COPD during a 5-year follow-up period. A total of 302 male patients aged >55 years were allocated to PPSV23, PCV13, and vaccine naïve groups. The primary endpoints were defined as the frequency of pneumonia and COPD exacerbations per year, whereas the secondary endpoint included indicators of disease severity, such as the BODE index, MMRC and the CAT index, FEV1 and the 6-min walk test. After 1 year, the frequency of having at least one pneumonia reduced to 4.9% in PCV13, and to 6.3% in the PPSV23 groups versus 15% in the control group. However, this efficacy was significantly reduced from the second year of the follow-up: the patients with PPSV23 showed a higher pneumonia rate compared to both PCV13 and control patients. By year 5, 47% of the PPSV23-vaccinated patients developed pneumonia, compared to 3.3% in the PCV13 (*p* < 0.001). Regarding exacerbations, the results were similar: COPD exacerbations had a frequency of 81.3% in the PPSV23 group, as compared to 23.6% in the PCV13 patients (*p* < 0.001). Although in the first year both vaccines caused a protective effect against exacerbations, from the second year, the PPSV23 group showed a gradual decline, reaching the level of the control group by year 5 post-vaccination. The trial demonstrated that PCV13 is associated with long-term protection, lasting at least 5 years, against episodes of pneumonia or COPD exacerbations. PPSV23-vaccinated patients are at an increased risk of having pneumonia or exacerbations, similar to the vaccine naïve patients [56].

In recent years, there has been a need to develop novel PCV vaccines, as new *S.pneumoniae* serotypes appeared to cause pneumococcal diseases, and antibiotic resistance was also associated with these serotypes [66,67]. In 2021, a 20-valent (PCV20) and a 15-valent (PCV15) pneumococcal vaccine were licensed for adults aged >18 years and were included in the recommendations for all adults aged >65 years and for 19–64-year-old adults with underlying medical conditions or risk factors who had not received the PCV vaccine before [68].

PCV15 contains the 22F and 33F serotypes, in addition to PCV13, demonstrating comparable immunogenicity and safety profile in adults aged >50 years. Prior to and 1 month post-vaccination, PCV15 induced serotype-specific antibodies to a similar or higher extent than PCV13. In parallel, these antibodies showed functionality and reached comparable opsonophagocytic activity (OPA) [69]. PCV20, which contains 8, 10A, 11A, 12F, 15B, 22F, and 33F serotypes in addition to the PCV13 strains, elicited favourable safety profile and robust serotype-specific responses, such as OPA, both in young adults and the elderly [70,71].

### 3.6. Co-Administration of PCVs and Antiviral Vaccines

The co-administration of pneumococcal conjugate vaccines and other vaccines such as the influenza vaccine was also assessed. A clinical trial hypothesised that the immune responses to PCV13 and quadrivalent inactivated influenza vaccine (QIV) in older adults can be influenced by the co-administration or by a past PPSV23 vaccine. An overall 882 ≥ 50 years old patients pre-immunized with ≥1 dose of PPSV23 ≥ 1 year before enrolment received PCV13 + QIV, then placebo 1 month later or placebo + QIV, then PCV13 1 month later. Of 882 patients, 846 showed immunogenicity, and the immune responses to pneumococcal strains and all four influenza strains were comparable in both groups. the co-administration of PCV13 and the influenza vaccine was noninferior, as compared to the immune responses to vaccines given alone. PCV13 can be administered safely concomitantly to PPSV23-pre-immunized adults [72].

In a recent phase 3, randomized, double-blind, multicentre study enrolling 1796 elderly (>65 years of age), participants received either PCV20 and QIV, followed 1 month later by saline, or QIV and saline, followed 1 month later by PCV20. In terms of antibody responses, the groups did not show significant differences in OPA titers for PCV20 and strain-specific hemagglutination inhibition assay (HAI) titers for QIV. Co-administration of PCV20 with QIV was safe and tolerable, with only mild or moderate fatigue being observed frequently. The safety profile and immunogenicity of PCV20 was similar, either given alone or in combination, so the co-administration of PCV20 and QIV can be supported [73].

As COVID-19 vaccination increased in recent years, it became clear that the possibility of co-administration of the BNT162b2 COVID-19 vaccine and the PCV20 vaccine may prevent elderly or adults with chronic conditions from developing severe pneumonia and pneumococcal diseases. A randomized, phase 3 study evaluated the safety and immunogenicity of the co-administration of PCV20 and the third dose of BNT162b2 COVID-19 vaccine. The trial recruited 570 participants aged ≥65 years, who randomized to PCV20 and BNT162b2 co-administered, or PCV20, or BNT162b2-only groups. The results suggested that the neutralizing titers against SARS-CoV-2 virus and the S-binding IgG titers were similar in the co-administration group and the BNT162b2-only group. Similarly, the immunogenicity of PCV20 was comparable, showing noninferiority if it was co-administered, as compared to participants receiving PCV20 only [74].

Based on these data, there are still ongoing studies to extrapolate the efficacy of co-administered COVID-19 and PCV vaccines. Currently, data are scarce or not available on the co-administration of PCV15 or PCV20 with other vaccines, such as pertussis, tetanus, diphtheria, or hepatitis B among adult patients with underlying conditions.

Currently, both PCV15 and PCV20 are included in the recommendations of CDC against pneumococcal diseases: PCV20 alone or PCV15 in series with PPSV23 are promoted in adults aged >65 years and in 19–64-year-old adults with risk factors [68]. As a result, GOLD 2023 incorporated the recommendation of PCV15, PCV20, and PPSV23 in the immunization program of patients with COPD, suggesting the co-administration with influenza vaccine [1,75].

**Figure 1:**
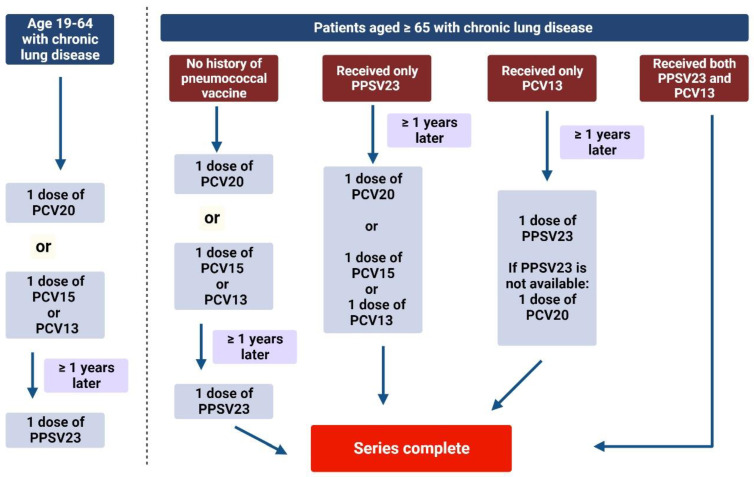
Recommendations for the pneumococcal vaccination for COPD patients. The protocol can be dependent on the vaccine availability in each country. Vaccination recommendation is based on CDC guidelines [12]. PCV: pneumococcal conjugate vaccine; PPSV23: pneumococcal polysaccharide vaccine.

The current recommendations for the use of pneumococcal vaccines in COPD are summarised on Figure 1.

## 4. Main Virulence Factors of *S. pneumoniae* and Potential Future Vaccine Targets

There is emerging evidence that non-vaccine, unencapsulated serotypes of *S. pneumoniae* are related to COPD exacerbations and invasive pneumococcal diseases [76]. Patients with COPD present an altered and decreased epithelial defence mechanism, such as increased permeability of the airway epithelium, impaired ciliary function, reduced mucociliary clearance, increased mucus production, reduced secretory IgA production, and altered neutrophils and alveolar macrophage functions. This epithelial barrier dysfunction increases the chances of pathogens to enter and cause further changes in host defence responses [77]. Thus, new vaccine development approaches include vaccine candidates against pneumococcal proteins via new protein antigens or genetic manipulations of the main pneumococcal proteins [25].

The surface proteins of *S. pneumoniae*, pneumococcal surface protein A (PspA) and choline-binding protein A (CbpA, previously known as pneumococcal surface protein C, PspC) play an important role in inhibiting complement-mediated killing and opsonophagocytosis. PspA can bind directly to factor B and accelerate the dissociation of C3 convertase or block the enzyme directly, thus changing the alternative pathway to eliminate the bacteria [78]. PspA has the ability to interfere with the host’s Fe-free form of lactoferrin, resulting in the avoidance of bacteria killing [79]. CbpA can also influence host complement activation via binding directly to C3 and factor H, decreasing the complement deposition of C3, and in some *S. pneumoniae* strains, having an interaction with the pathway inhibitor C4b-binding protein (C4BP). As a consequence, these surface proteins can be highly immunogenic, representing a suitable target for further vaccine development. Preliminary results of murine models confirmed that the vaccine against PspA induced sufficient antibody response, and the levels of anti-PspA IgG in the offspring sera derived from immunized mothers was significantly higher compared to those in the offspring from sham-immunized mothers [80,81]. A recombinant PspA-based vaccine (PspAB1-5) showed efficacy in modifying complement-mediated killing through the deposit of more C3 complement components on the surface of the *S. pneumoniae* strain [82]. Similar to PspA, PspC was investigated as a vaccine target due to its high immunogenicity and ability to influence the complement system. However, this surface protein is highly variant in pneumococcal strains, and this diversity reflected functional differences. This phenomenon ensured an escape benefit in the presence of anti-PspC variant-specific immunity; thus, PspC is not a suitable vaccine target [83].

Promising results were published against pneumolysin (Ply), which is responsible for lytic activity, complement activation, and depletion via the Toll-like receptor-4 (TLR4) and pyrin domain-containing protein-3 (NLRP-3) inflammasome [84]. A multi-antigen vaccine, PnuBioVax (PBV), contains PspA, Ply, and pilus-1 subunits to induce serotype-independent immunity and has been investigated in immunised rabbits. Sera from PBV-immunised rabbits contained high levels of IgG antibodies against PspA, Ply, PsaA, and PiuA, and demonstrated enhanced opsonophagocytic killing [85]. In a phase 1 study, 36 healthy adults (18–40 years) were randomised to receive three different doses of PnuBioVax (50, 200, 500 µg) or placebo. Volunteers receiving 200 and 500 µg of PnuBioVax demonstrated at least a two-fold increase in antibodies against Ply, PspA, and pilus proteins (RrgB and RrgA). The increase was significantly higher than in subjects receiving 50 µg PnuBioVax or placebo. Regarding safety, all doses were well-tolerated [86]. Since then, fusion proteins including Ply and other candidates, such as a lipoprotein SP0148, showed high immunogenicity in murine models, caused significantly lower lung bacterial loads, attenuated lung inflammation, and was associated with strong Th1, Th2, and Th17 cell responses against *S. pneumoniae* [87]. Similar results were found with a PspA-Ply fusion protein-based vaccine in mice: a significant production of TNF-α and IL-6 in the bronchoalveolar lavage fluid (BALF) was observed, correlating with protection against pneumonia [88].

Pneumococcal histidine triad protein D (PhtD) has an important role in Zn acquisition, thus influencing the host cell Zn metabolism and its immune response to bacteria [89]. In a mouse model, intranasal PhtD-based vaccine induced high serum antibody and CD4 Th1-biased immune memory and triggered protection against pneumococcal colonization [90]. In a phase I trial, healthy adults aged 18–50 years were recruited and received an intramuscular injection of either 6, 25, or 100 μg of the PhtD vaccine. The recombinant PhtD-based vaccine showed increased antibody titres without serious adverse events [91]. Another phase I/II PhtD-based vaccine clinical trial included 150 older (>65 years old) and 147 young (18–45 years old) adults. The subjects were randomised to receive two doses (months 0 and 2) of PhtD 30 μg, PhtD 10 μg plus alum, PhtD 30 μg plus alum, PhtD 10 μg plus AS02V or PhtD 30 μg plus AS02V, or the 23-valent polysaccharide pneumococcal vaccine (23PPV) at month 0, with placebo at month 2. The results showed that in older participants, anti-PhtD titres were lower than in young subjects, but the use of the adjuvant AS02V with PhtD 30 μg induced an increased frequency of PhtD-specific CD4 cells and higher memory B cell responses in both age groups [92]. A trivalent protein-based vaccine containing recombinant PcpA, PhtD, and PlyD1 showed safety and a higher than two-fold increase in antibody titres against the three proteins in children and adults in a phase I human study [93]. A phase II trial including 156 adults investigated the formulation carrying the mixture of PhtD, pneumolysin toxoid (dPly), and the 10-valent pneumococcal non-typeable *H. influenzae* protein D conjugate (PHiD-CV) vaccine and confirmed that dPly and PhtD alone or in combination with PHiD-CV were immunogenic and well-tolerated [94].

Other vaccine targets were also analysed, such as the elongation factor TU (EF-Tu), which is released through the autolysis of *S. pneumoniae* and can induce the production of a range of pro-inflammatory cytokines. EF-Tu can be present on the cell surface; thus, it can be a novel target for vaccine development. Animal studies showed that a recombinant EF-Tu-based vaccine triggered the production of inflammatory cytokines, the IgG1 and IgG2a antibodies, and enhanced the phagocytic activity of macrophages against *S. pneumoniae* infection, independently of the serotypes [95]. Novel studies also suggested that EF-Tu could be a vaccine candidate, particularly in combination with neuraminidase A (NanA) and Ply. A study tested a novel fusion protein, NanAT1-TufT1-PlyD4, and found that it reduced the *S. pneumoniae* lung colonization and induced Th1, Th2, and Th17 responses in the host [96].

In addition, there are several ongoing studies to develop vaccines based on diminishing autolysin (lytA) or using genetically modified live attenuated whole cell vaccines or to create nanovaccines [97,98,99].

**Figure 2:**
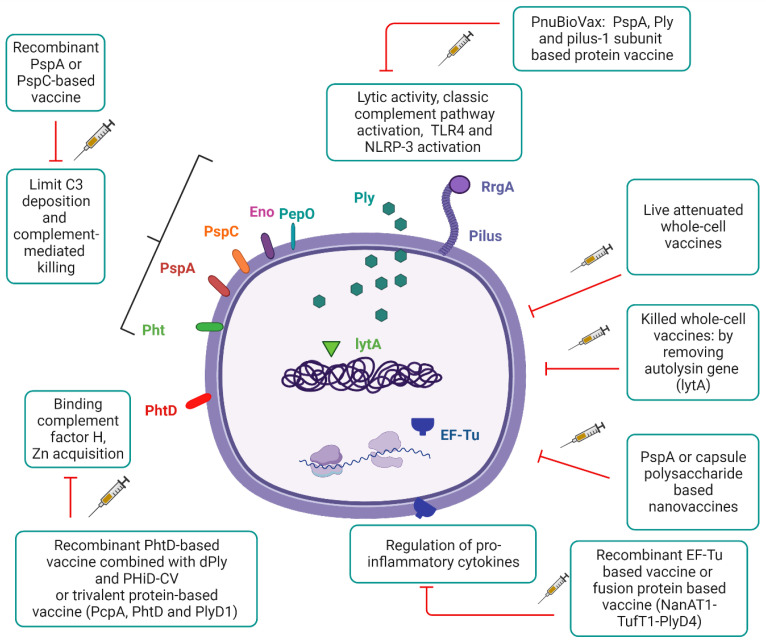
Key potential virulence factors as pneumococcal vaccine targets.

The main virulence factors that can be potential vaccine candidates are shown on Figure 2.

Pneumolysin (Ply) is a pore-forming toxin that activates TLR-4 and NLRP-3, induces the production of pro-inflammatory cytokines, and interacts with the complement system. Autolysin can facilitate the release of Ply, and its gene (lytA) is removed to develop killed whole cell vaccines. The pneumococcal proteins, such as pneumococcal surface protein A (PspA), choline-binding protein A (CbpA, also known as PspC), enolase (Eno), and pneumococcal histidine triad protein (Pht) can prevent complement activation and deposition. Endopeptidase (PepO) can inhibit the classical pathway of the complement system by binding to C1q. Currently, there are several recombinant protein-based vaccines or nanovaccines containing PspA in combination with Ply or other structural components. In the case of piliated strains, the pilus subunit tip adhesins, such as RrgA, can also serve as a potential antigen target. The elongation factor Tu (EF-Tu) is one of the most conserved in *S. pneumoniae*, having chaperone activity during protein synthesis; thus, recombinant EF-Tu could be a novel antigen candidate for future vaccine development.

## 5. Emerging Bacteria in COPD Exacerbations and Their Prevention

In addition to *S. pneumoniae*, the major bacterial causes of COPD exacerbations include non-typable *H. influenzae* (NTHi), *M. catarrhalis* and *Pseudomonas aeruginosa* [100,101,102]. Approximately half of the bacterial exacerbations are induced by NTHi, this bacterium being the most common in the respiratory tract in this population [103]. NTHi strains are not encapsulated; thus, a surface antigen that can be targeted for immunization is lacking. The first attempts to develop a vaccine against NTHi contained lipooligosaccharide (LOS) without O-antigen, but it proved to be toxic and deemed to be useless for immunization [104]. In the first trial, 48 healthy adults aged 18–40 years received two vaccine formulations (PD and a fusion protein PE-PilA); in the second study, 270 current or former smokers, aged 50–70 years received vaccine formulations. All formulations were well-tolerated, had acceptable safety profiles and triggered robust immune responses in adults. Based on these data, further clinical trials were conducted with the NTHi vaccine against protein D, a fusion-protein consisting of PilA and protein E [105].

*M. catarrhalis* can be a co-pathogen in the respiratory tract, promotes the survival of NTHi, and increases antibiotic resistance [106,107]. Based on a retrospective study, from sputum samples in which *M. catarrhalis* can be detected, 26.7% of isolates contained also NTHi [101]. Thus, it was hypothesized that combined vaccination may bring benefits in COPD patients through the prevention of exacerbations [108].

Damme et al. analysed the tolerability, safety, and immunogenicity of vaccine formulations containing surface proteins of NTHi (PD and PE-PilA) and *M. catarrhalis* ubiquitous surface protein A2 (UspA2) in adults with a smoking history ≥10 pack-years, representing the COPD population.

Patients received two doses with a 2 month-interval in a randomised, observer-blind, placebo-controlled study, demonstrating good humoral immune responses against NTHi and *M. catarrhalis* across groups. These data suggest that the *NTHi–M. catarrhalis* vaccine can be a reasonable tool for the prevention of COPD exacerbations [109].

A phase 2-controlled trial assessing immunogenicity involved 145 patients with moderate/severe COPD who were randomized and treated with two doses of NTHi vaccine containing recombinant protein D, protein E, and Pilin A (PE-PilA), as well as an AS01 adjuvant. Anti-PD, anti-PE, and anti-PilA antibody concentrations increased up to 30 days after each NTHi vaccine dose and maintained higher levels than baseline up to 1-year post-vaccination. Although immune responses were satisfactory, there were no statistically significant changes in the frequency of exacerbations (the yearly rate was 1.49 for the vaccinated group versus 1.73 for the control group) [110].

A similar tendency was observed in another multicentre, randomised, placebo-controlled phase 2b trial that evaluated the effects of NTHi-Mcat-combined vaccination in patients with COPD. In total, 606 participants were recruited, with 304 in the NTHi-Mcat group and 302 in the placebo group. After receiving two doses, one-year post-vaccination, the exacerbation rate was assessed. The results were disappointing, showing an average exacerbation yearly rate for NTHi-Mcat group of 1.22 versus 1.17 in the control group: the vaccine was ineffective (*p* = 0.82). No significant differences were observed between the groups in time to first exacerbation (*p* = 0.58). In a post-hoc analysis where patients were categorised by blood eosinophil count and inhaled corticosteroid use, there were no significant reductions in exacerbation rates in the vaccinated group. The results confirmed the vaccine’s immunogenicity and safety, but not its clinical effectiveness in reducing COPD exacerbations [111].

Whooping cough caused by *Bordetella pertussis* is still underdiagnosed in the context of the elderly and patients with risk factors. Adults aged >65 years are at a 4–6 times higher risk of being hospitalized because of pertussis. Therefore, the assessment of risk and pertussis prevention in COPD can be crucial [112]. It is also well-known that after the last dose of Tdap, its effect lasts between 5 and 12 years, and booster vaccinations are needed every 10 years to maintain immunity [113].

In an observational study involving 104 patients, aged 40–85 years, with moderate/severe COPD, testing for pertussis-specific antibodies (anti-PT) revealed that 14 out of 104 (13.5%) patients exhibited anti-PT concentrations ≥50 IU/mL in either year 1 or year 2. Moreover, 5.8% of patients demonstrated anti-PT levels ≥70 IU/mL, indicating the presence of *B. pertussis* circulation within this population, and underscoring the importance of considering vaccination in COPD [114]. A retrospective study further confirmed that pertussis has a higher prevalence among individuals with pre-existing respiratory conditions such as COPD and asthma, thereby imposing an increased economic burden on healthcare services [115]. Tdap demonstrates a favourable safety profile and immunogenicity across all adult age groups (>19 years), with recommendations for administration every 10 years as a booster to ensure sustained protection [116]. The Global Initiative for Chronic Obstructive Lung Disease (GOLD) has incorporated the Centers for Disease Control and Prevention (CDC) recommendation for Tdap vaccination to safeguard against pertussis, tetanus, and diphtheria in individuals who did not receive vaccination during adolescence [1]. Despite the implementation of routine booster vaccination in several European countries, prospective trials are still required to evaluate vaccine efficacy in preventing COPD exacerbations [117].

## 6. Conclusions

Patients with COPD are especially vulnerable to bacterial infections, which are the main causes of exacerbations and mortality. Vaccines can reduce the infective burden and could help maintain a stable COPD. Despite the increased risk of these patients, there is still insufficient vaccine coverage and vaccine awareness. This review highlighted the current state of vaccine recommendations against *S. pneumoniae* and other bacteria, such as *H. influenzae.* Recently developed new PCV vaccines are included in international guidelines, but despite the introduction of pneumococcal vaccines, *S. pneumoniae* serotypes that are not included in vaccine formulation have increased the prevalence of pneumococcal disease. We cannot be complacent, and further vaccine developments are needed to prevent pneumococcal disease and bacterial exacerbations in patients with chronic lung diseases.
